# Coronavirus disease (COVID-19) Community Testing Team in Scotland: A 14-day review, 6 to 20 February 2020

**DOI:** 10.2807/1560-7917.ES.2020.25.12.2000217

**Published:** 2020-03-26

**Authors:** Kate Mark, Katie Steel, Janet Stevenson, Christine Evans, Duncan McCormick, Lorna Willocks, Alison McCallum, Laura Jones, Ingolfur Johannessen, Kate Templeton, Oliver Koch, Claire Mackintosh

**Affiliations:** 1 NHS Lothian Directorate of Public Health, Edinburgh, Scotland; 2 NHS Lothian Analytic Services, Edinburgh, Scotland; 3 NHS Lothian Paediatric Services, Edinburgh, Scotland; 4 NHS Lothian Virology Services, Edinburgh, Scotland; 5 NHS Lothian Regional Infectious Diseases Unit, Edinburgh Scotland

**Keywords:** COVID-19, community, containment, sampling

## Abstract

In response to the outbreak of COVID-19, we set up a team to carry out sampling in the community. This enabled individuals to remain in self-isolation in their own homes and to prevent healthcare settings and services from being overwhelmed by admissions for sampling of suspected cases. There is evidence that this is a cost effective, safe and necessary service to complement COVID-19 testing in hospitals.

The outbreak of respiratory illness, now known as coronavirus disease (COVID-19), caused by an unknown pathogen subsequently identified as severe acute respiratory syndrome coronavirus 2 (SARS-CoV-2) was first reported in Wuhan, China on 31 December 2019 [1]. Subsequently, SARS-CoV-2 affected other parts of the world, with imported cases of infections detected the United Kingdom (UK) in January 2020 [2]. Up to 12 March 2020, the strategy in the UK was to contain the spread of the disease, with suspected cases of COVID-19 referred for clinical assessment and rapid SARS-CoV-2 testing. Sampling of suspected COVID-19 cases requires strict infection control precautions, in an environment that does not risk contamination of healthcare settings. To address this challenge, sampling in the community where possible can contribute to limit the spread of COVID-19 [3]. This report presents community sampling for SARS-CoV2 testing in the Lothian region of Scotland, UK.

## Protocol development

In Lothian, a region hosting several universities including a large population of domestic and international students, health services are provided by the National Health Service (NHS) Lothian, which is led by the NHS Lothian health board. All together, the board serves ca 900,000 people. Cases for COVID-19 are classified according to Health Protection Scotland (HPS) [4] and Public Health England (PHE) [5] definitions, which correspond to those of the European Centre for Disease Prevention and Control (ECDC) for suspected, probable and confirmed cases [6], used in this study. 

Before 12 March 2020, all persons meeting the suspected case definition in Lothian were referred for SARS-Cov-2 infection testing. To enable sampling in the region’s community, a number of departments within NHS Lothian provided expertise and support. Public Health and Health Policy, Regional Infectious Diseases Unit (RIDU) and Primary Care collaborated to develop a protocol, which was agreed with NHS Lothian leads in Infection Control, Waste Facilities and Transport. We also consulted HPS.

A COVID-19 Community Testing Team was staffed by consultants and training grade doctors from RIDU, adopting a ‘train the trainer’ approach [7] with nurses, to enable them to conduct sampling in the community. A dedicated ambulance car and driver were seconded from the out of hours general practice (GP) service. NHS Lothian Health and Safety prioritised fit testing for filtering face piece (FFP)3 masks for personnel involved to ensure appropriate personal protective equipment (PPE) could be used. 

As no guidance on donning and doffing PPE in the community was available, it was decided that the team should develop a risk assessment of the environment before attending, to ensure this could be done safely, either outside the property, or in a space identified inside such as porch, and with discretion as required. 

Moreover, because suspected cases needing further care in the hospital should be tested there, cases to be sampled by the COVID-19 Community Testing Team would have to be fit enough to remain at home. Therefore two consecutive assessments by telephone were to be conducted prior to referral to the team, first by a GP to assess if it was clinically appropriate for a person to remain at home, then by RIDU or Public Health and Health Policy staff to ensure if it was suitable to test this patient according to case definition. 

The COVID-19 Community Testing Team was mobilised within three working days and commenced its work on 6 February 2020. An iterative approach to assessment and sampling in the community was encouraged to enable learning for the team, and across Scotland.

## Epidemiology

Between 6 February and 20 February 2020, the health board was notified of 94 potential cases of COVID-19. Of the 94, 85 met the case definition for ‘suspected cases’. The COVID-19 Community Testing Team sampled 79 of these. During the same period, the six remaining suspected cases were sampled in a hospital setting. 

**Figure 1:**
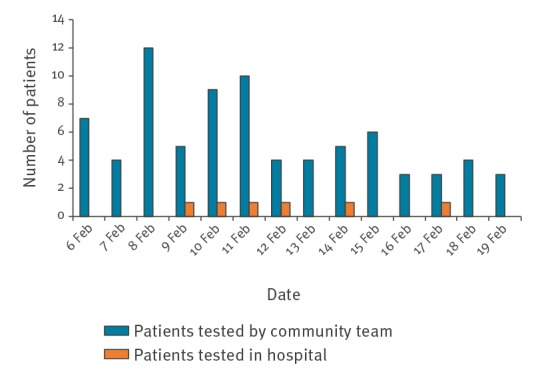
Distribution of the number of suspected cases of COVID-19 sampled in NHS Lothian health board over a 14-day period, by location of sampling, Scotland, United Kingdom, 6–20 February 2020 (n = 85 suspected cases)

COVID-19: coronavirus disease; NHS: National Health Service.

For the 79 cases in the community, the median time from referral to test was 1 day, and the maximum time was 3 days. A total of 76 cases (96.2%) were sampled the same day or the following day after notification. The mean number of samples in the community taken in a day was 6.1 with a maximum of 11 samples gathered in 1 day. The testing capacity of the laboratory for East of Scotland, including Lothian, was 100 samples per day. Figure 1 shows the distribution of 85 suspected cases sampled in Lothian.

**Figure 2:**
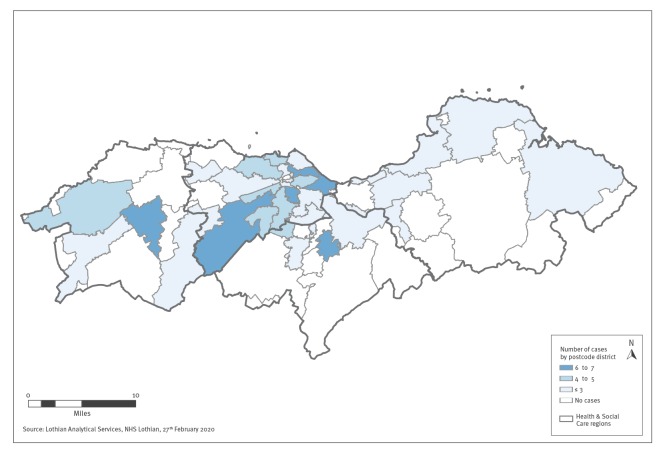
A heatmap showing distribution of suspected cases sampled by COVID-19 Community Testing Team across Lothian, Scotland, United Kingdom, 6–20 February 2020 (n = 79 cases)

The age range of the 79 suspected cases sampled by the community testing team was 1–77 years and 36 (45%) were male. A total of 51 (64.6%) lived in City of Edinburgh Health and Social Care region, and approximately one third lived outside the city. Figure 2 demonstrates a heatmap of the distribution of suspected cases sampled by the community testing team across Lothian.

COVID-19: coronavirus disease.

Of the six suspected cases who were sampled in hospital over the study period, two were aged under 2 years old and required testing by a paediatrician. The COVID-19 Community Testing Team later included a paediatrician as part of the team, to prevent children requiring hospital sampling where possible. The other four cases had a more complex presentation and required further assessment. 

## Patient pathways

Prior to the commencement of the COVID-19 Community Testing Team on 6 February 2020, 11 suspected cases were notified to NHS Lothian Health Protection Team. These all required transfer to a hospital setting for COVID-19 using special arrangements by ambulance. The different pathways for sampling a suspected case are outlined in the Table, with the associated estimated costs, and other relevant factors when considering sampling of high consequence infectious disease (HCID).

**Table:** A narrative summary comparing pathways and costs^a ^for sampling for suspected cases of COVID-19, Scotland, February 2020

Criteria	Community sampling	Specialist ambulance and hospital sampling	A&E attendance and hospital sampling^b^
**DIRECT COST**	Driver: GBP 84 (EUR 93) per day. 2 agency nurses: GBP 305 (EUR 348) per day. 1 agency doctor and 1 agency nurse at weekend: Additional GBP 242 (EUR 276) per day at weekend.	Specialist isolation ambulance transfer and attendance at IDU: GBP 768 (EUR 860) per patient.	Side room in A&E and unplanned attendance: GBP 186 (EUR 212) for category 3 triage per patient
	No overnight stay required^c^.	May require overnight stay in RIDU: GBP 687 (EUR 784).	May require stay in A&E or MAU side room. 12 hour stay in MAU side room: GBP 350 (EUR 400)
	Approximate cost per patient: GBP 55 (EUR 61).	Approximate cost per patient with overnight stay: GBP 1,455 (EUR 1,626).	Approximate cost per patient with stay in MAU: GBP 536 (EUR 591).
**HEALTHCARE TIME PER PATIENT**	Up to 1 hour travel time per patient.	Transport time very variable, can be between 1 and 6 hours^d^.	Transport time variable depending on mode of arrival.
	Approximately 30 min face to face with patient.	Face to face with patient up to 1 hour.	Face to face with patient up to 1 hour.
	No overnight stay required^c^.	Overnight stay may be required.	Overnight stay may be required.
	No decontamination required.	Decontamination of ambulance and room required. Can take up to 6 hours.	Decontamination of all areas patient was located. Can take up to 6 hours.
**HEALTHCARE CONTACTS AND EASE IN WHICH THESE CAN BE IDENTIFIED**	2 staff trained in PPE use. Straightforward to identify if required.	2 paramedics. At least 2 staff from RIDU (may be more if longer stay due to awaiting required transport^e^ or timing of attendance). Straightforward to identify if required.	Multiple healthcare staff from all professions. Challenging to identify rapidly if required.
**PUBLIC CONTACTS**	Household only. No new public contacts.	Household only. No new public contacts.	Multiple contacts.
**IMPACT**	Low risk to patient, staff and public.	Low risk to patient, staff and public.	Low risk to patient. Higher risk to staff and public.
	Low risk to healthcare system. Small numbers of staff required. Low risk of contamination.	Medium risk to healthcare system. Small numbers of staff but highly trained and limited resource of specialist ambulance crew. Opportunity costs of use of healthcare service for clinically stable patients. Low risk of contamination.	High risk to healthcare system. Requires resource of side rooms in acute settings. No additional staff required however, high risk of staff exposure and subsequent staff exclusion if required. High risk of contamination of multiple places of care.

A&E: Accident and Emergency Department; ID; Infectious Diseases; MAU: Medical Admissions Unit; NHS: National Health Service; PH: Public Health and Health Policy; PPE: personal protective equipment; RIDU:  Regional Infectious Diseases Unit.

^a^ Costs are approximate from NHS Lothian Finance Department and Scottish Ambulance Service Procurement. GBP to EUR conversion calculated on 11 March 2020.

^b^ Despite not being advised, the event of this option being taken by a COVID-19 case cannot be completely eliminated.

^c^ The testing team only attends patients well enough to be at home. Before sampling in the community is carried out, patients are assessed by telephone by the general practice services for clinically appropriateness to remain home. A second assessment is done by RIDU or PH staff by telephone to check if they meet the case definition for testing.

^d^ Transport times are highly variable as dependent on limited resource of specialist ambulance crew, and distance of person to the infectious disease unit. In contrast, community teams are able to attend at person’s home so no patient travel time, and they are usually within 1 hour of person’s address.

^e ^The patient may need to stay longer in the hospital while waiting for the ambulance to be available/ready to take him/her back home.

The cost of the specialist ambulance and hospital pathway for management of suspected cases of COVID-19 could be up to GBP 1,455 (EUR 1,626) if an overnight stay was required. This was the recommended pathway when a suspected case of COVID-19 first presented in Scotland. Even if the 79 cases did not require an overnight stay, the estimated cost of ambulance transfer alone would have been GBP 60,672 (EUR 66,283). The direct costs of the community testing service include cost of a driver, bank staff nurses, and locum payments for additional medical shifts at weekends. This was a total of GBP 4,374 (EUR 4,779) over 14 days, and GBP 55 (EUR 60) per patient.

Beyond the direct financial costs, there are other obvious benefits to community sampling. It limits the exposure of healthcare workers, reduces PPE requirement for multiple members of staff, reduces the burden of decontamination procedures and protects the public from being unnecessarily exposed to a suspected case of COVID-19. It also prevents essential clinical services being diverted from providing care to patients with clinical need.

## Discussion

This study offers an example of how sampling COVID-19 suspected cases in the community can be applied. In the COVID-19 suspected case definition, several scenarios based on whether local transmission is occurring are considered. If local transmission is not taking place, epidemiological criteria, such as a history of travel or residence in a country/area reporting local or community transmission may be part of the suspected case definition. In Scotland, the first suspected case of COVID-19 was identified on 23 January 2020 [8]. It was evident that the ambulance services and the hospitals could be rapidly overwhelmed, particularly if the case definition widened, due to the consideration of more geographical areas abroad with local transmission, or if local transmission would begin in Scotland itself, leading to dismissal of epidemiological criteria all together. This was evident when HPS and PHE adopted a broader number of countries in the case definition than that used by ECDC on 25 February 2020. The threat of COVID-19 has required healthcare services to react and adapt their working patterns. There was no available guidance or similar service in Scotland yet when the COVID-19 Community Testing Team was initiated. Other services were in development across the UK, but these were also in their infancy [9].

Self-isolation of suspected cases and avoiding healthcare settings where feasible, is fundamental to limiting potential spread of the virus. The risk of a ‘super-spreading event’ [10] occurring in a healthcare setting should be minimised as far as possible. In order to detect and control an outbreak, community sampling should be a priority. The COVID-19 Community Testing Team quickly identified that sampling in patients’ homes was a necessary service to ensure rapid testing and safe containment. The Chief Medical Officer in Scotland stipulated in a communication on 13 February 2020 [11] that all suspected cases of COVID-19 should be sampled within 24 hours of presentation. Our approach achieved this target in over 95% of suspected cases. Furthermore, suspected cases were able to stay within their own homes with a clear management plan and rapid sampling as well as receiving reassurance from an experienced clinical team.

Prior to the inception of the COVID-19 Community Testing Team on 6 February 2020, a suspected case was transported to a specialist Infectious Diseases Unit by special arrangement with the Scottish Ambulance Service (SAS) [12], regardless of clinical need. This is a national resource reserved for transportation of suspected HCIDs and is thus limited. Prioritisation of the use of this service depends on clinical need and thus there was often a delay before transport could be arranged. As at 5 February 2020, 27 suspected cases had been tested in Scotland, with more awaiting testing. This meant a considerable strain on the ambulance service. Once used, the ambulance and examination room within the hospital both required terminal cleaning and decontamination following use by a single patient, which further limited resources available. This route was expensive, resource intensive, added pressure to a hospital system already under strain from increased winter admissions. The limited capacity due to the surge of cases in the RIDU led to patients with complex infections being unable to access admission for specialised services. Ultimately, the process for sampling was highly disruptive and difficult, especially for clinically stable cases who nevertheless had to face a lengthy wait and exposure to a highly medicalised setting. Many suspected cases of COVID-19 had mild symptoms and did not require hospital assessment or admission. Patients were triaged by their GP service, and a specialist in Infectious Diseases or Public Health reviewed referrals. Community sampling enabled the ambulance service to have more capacity to respond to clinical urgent cases, and ensured inpatient beds were available for those in need.

A notable challenge we faced was limited staff resource. Senior clinicians in RIDU were quick to recognise the necessity of this service to prevent primary and secondary care healthcare settings from becoming overwhelmed. They led and performed the sampling in the initial weeks. However, this was not sustainable due to other work pressures. Although nursing staff were encouraged to provide this service, identifying staff, providing training, and arranging timely fit testing was difficult. The COVID-19 response requires an oversight of the impact on services and the ability to rapidly deploy staff in areas as required, while ensuring staff and public safety.

There is a relative lack of guidance in infection prevention and control for donning and doffing PPE in the community. We took a pragmatic approach and carried out a risk assessment of each suspected case’s home setting to assess the feasibility of safely donning and doffing at the case’s address. We received no media attention while carrying out sampling.

The daily capacity of the service was limited by travel time, as we covered a large geographical area, with often heavy traffic. Subsequent to this study, we developed a ‘drive through’ service for those able to access private transport safely. This greatly increased capacity of the service with 313 individuals sampled at the ‘drive through’ in a 14-day period between 28 February and 13 March 2020. The original Community Testing Team remained in situ during this period to sample individuals who could not attend the drive through service. We recommend that other services review their population to understand which model is most appropriate. Our population included a large number of students who do not have access to private transport and so a fixed site was not chosen as the initial model. 

Our model for community testing was adopted across other health boards in Scotland, and informed national guidance on community testing. It met the target time period between presentation and sampling set by the Scottish Government. It avoided significant expense of unnecessary admissions. Most importantly, it was successful in avoiding a large number of resource intensive hospital admissions for testing for SARS-CoV-2 that could have put the healthcare system at risk of exposure to COVID-19. Community sampling is an intrinsic part of the response to COVID-19 and can be carried out safely, efficiently and effectively. However, after further widening of the suspected cases definition, which accounted for an increase of geographical areas presenting risk for potential exposure abroad by HPS and PHE [5], the Lothian team witnessed a surge of referrals. Indeed, following the case definition widening on 25 February 2020, 49 suspected cases were referred for sampling in 48 hours, compared with 79 over the 14-day period in this study (6–20 February 2020). To meet this increased demand, we adapted our model to a fixed site, or ‘drive through’. This commenced on 28 February [13]. Local transmission of SARS-CoV-2 in Scotland and the UK nevertheless started with as at 19 March 2,644 confirmed cases in UK [14]. One possible reason may have been the mild course of COVID-19 in a relatively large proportion of those infected [15], so that such people unknowingly contributed to ongoing transmission. Facing the larger local spread of disease, the UK moved from applying containment to delay and mitigation measures [16]. Moreover, due to capacity reasons, testing could no longer be offered to people with minor symptoms from 12 March onwards [17]. All this may have affected the approach to community testing. Measures to increase testing capacity are nevertheless currently being taken [18]. Healthcare services, and the UK and Scottish Governments, must recognise the importance of maintaining isolation of suspected cases and avoiding unnecessary contact with healthcare settings, and direct resource into facilitating community testing as far as possible. Healthcare staff should have ongoing training in these areas, including PPE use, fit testing and community experience, in order to ensure staffing resilience in response to a HCID.

## References

[1] World Health Organisation (WHO). Coronavirus disease (COVID-19) outbreak. Geneva: WHO. [Accessed 24 Feb 2020]. Available from: https://www.who.int/emergencies/diseases/novel-coronavirus-2019

[2] European Centre for Disease Prevention and Control (ECDC). Communicable disease threats report. Week 5, 26 January-1 February 2020, Stockholm: ECDC; 2020. Available from: https://www.ecdc.europa.eu/sites/default/files/documents/communicable-disease-threats-report-1-february-2020.pdf

[3] Heymann DL Shindo N WHO Scientific and Technical Advisory Group for Infectious Hazards COVID-19: what is next for public health? Lancet. 2020;395(10224):542-5. 10.1016/S0140-6736(20)30374-3 32061313 PMC7138015

[4] Health Protection Scotland. Novel coronavirus (COVID-19) Guidance for health protection teams. Glasgow: HPS. [Accessed 24 Feb 2020]. Available from: https://hpspubsrepo.blob.core.windows.net/hps-website/nss/2935/documents/1_covid-19-guidance-for-hpt.pdf

[5] Public Health England (PHE). COVID-19: investigation and initial clinical management of possible cases. London: PHE. [Accessed 24 Feb 2020]. Available from: https://www.gov.uk/government/publications/wuhan-novel-coronavirus-initial-investigation-of-possible-cases/investigation-and-initial-clinical-management-of-possible-cases-of-wuhan-novel-coronavirus-wn-cov-infection

[6] European Centre for Disease Prevention and Control (ECDC). Case definition for EU surveillance. Stockholm: ECDC. [Accessed 24 Feb 2020]. Available from: https://www.ecdc.europa.eu/en/case-definition-and-european-surveillance-human-infection-novel-coronavirus-2019-ncov

[7] Yarber L Brownson CA Jacob RR Baker EA Jones E Baumann C Evaluating a train-the-trainer approach for improving capacity for evidence-based decision making in public health. BMC Health Serv Res. 2015;15(1):547. 10.1186/s12913-015-1224-2 26652172 PMC4676893

[8] British Broadcasting C**orpora**tion (BBC). China coronavirus: Two tested in Scotland given all-clear. London: BBC; 25 January 2020. Available from: https://www.bbc.co.uk/news/uk-scotland-51241245

[9] Whittington AM, Logan S, Goodman A, Houston A, John L, Wrigley F. Coronavirus: rolling out community testing for covid-19 in the NHS. BMJ blog. Feb 17^th^ 2020.

[10] Stein RA Super-spreaders in infectious diseases. Int J Infect Dis. 2011;15(8):e510-3. 10.1016/j.ijid.2010.06.020 21737332 PMC7110524

[11] Chief Medical Officer Scotland Letter. 13th February 2020. Available from: https://www.sehd.scot.nhs.uk/cmo/CMO(2020)1.2.pdf

[12] Health Protection Scotland. (2020) Novel Coronavirus Guidance for Secondary Care. Available from: https://hpspubsrepo.blob.core.windows.net/hps-website/nss/2936/documents/1_covid-19-guidance-for-secondary-care.pdf

[13] British Broadcasting Corporation (BBC) Scotland. Coronavirus: Drive through testing begins at Edinburgh hospital**.** 28 February 2020. Glasgow: BBC Scotland. Available from: https://www.bbc.co.uk/news/uk-scotland-51678008

[14] Johns Hopkins University (JHU). Coronavirus COVID-19 Global Cases by the Center for Systems Science and Engineering (CSSE) at Johns Hopkins University (JHU). Baltimore: JHU. Available from: https://www.arcgis.com/apps/opsdashboard/index.html#/bda7594740fd40299423467b48e9ecf6

[15] World Health Organisation (WHO). Situation Report 41. Geneva: WHO. Available from: https://www.who.int/docs/default-source/coronaviruse/situation-reports/20200301-sitrep-41-covid-19.pdf?sfvrsn=6768306d_2

[16] European Centre for Disease Prevention and Control (ECDC). Novel coronavirus disease 2019 (COVID-19) pandemic: increased transmission in the EU/EEA and the UK – sixth update – 12 March 2020. Stockholm: ECDC; 2020.Available from: https://www.ecdc.europa.eu/en/publications-data/rapid-risk-assessment-novel-coronavirus-disease-2019-covid-19-pandemic-increased

[17] British Broadcasting Corporation (BBC) Scotland. Coronavirus: Scottish Covid-19 testing expanded to communities. Glasgow: BBC Scotland; 15 March 2020. Available from: https://www.bbc.co.uk/news/uk-scotland-51895936

[18] United Kingdom Government. Testing for coronavirus (COVID-19) will increase to 25,000 a day. 18 March 2020. Available from: https://www.gov.uk/government/news/testing-for-coronavirus-covid-19-will-increase-to-25-000-a-day

